# Identification of compounds to promote diabetic wound healing based on transcriptome signature

**DOI:** 10.3389/fphar.2025.1576056

**Published:** 2025-06-02

**Authors:** Jiamin Shang, Zhaoyu Liu, Meidai Liang, Zijie Yin, Zeyu Yang, Xiaomeng Ye, Guanhua Du, Xiuying Yang

**Affiliations:** ^1^ Beijing Key Laboratory of Innovative Drug Discovery and Polymorphic Druggability Research for Cerebrovascular Diseases, Institute of Materia Medica of Peking Union Medical College, Beijing, China; ^2^ Department of Pharmacy, Beijing Tsinghua Changgung Hospital, School of Clinical Medicine, Tsinghua Medicine, Tsinghua University, Beijing, China

**Keywords:** diabetic wound, bioinformatics, transcriptome, drug discovery, mechanism

## Abstract

**Purpose:**

Diabetic wounds are characterized by delayed healing, and the resulting diabetic foot ulcer may lead to severe complications, including amputations and mortality. This study aimed to identify potential small molecule drug candidates that can enhance diabetic wound healing through integrating transcriptome signature and experimental validation strategies.

**Method:**

Gene expression dataset (GSE147890) from a diabetic skin humanized mice model in the Gene Expression Omnibus database was analyzed to identify differentially expressed genes between diabetic and normal skin, as well as the wound edge at 24 h. The DEGs were integrated with wound-related genes from the Comparative Toxicogenomics Database to construct a diabetes-specific wound gene profile. Then, the expression signatures were analyzed using the ClusterProfiler package in R for Gene Ontology and Kyoto Encyclopedia of Genes and Genomes (KEGG) enrichment analyses. Hub genes were identified through the String database and Cytoscope software. The Connectivity Map (CMap) was employed to predict compounds with potential therapeutic effects on diabetic wound healing. These predications were validated through *in vitro* and *in vivo* experiments.

**Results:**

A total of 167 DEGs were identified between diabetic and normal wounds, with significant enrichment in biological processes related to the extracellular matrix and collagen. The top ten hub genes were predominantly associated with collagen synthesis and inflammatory responses. CMap analysis identified 12 small-molecule compounds, top four of which were further investigated. *In vitro* experiments demonstrated that two compounds promoted fibroblast proliferation. *In vivo* studies revealed that compound CG-930 enhanced early inflammatory responses and upregulated the Nod-like receptor signaling pathway, significantly improving wound healing in streptozotocin (STZ) -induced diabetic mice.

**Conclusion:**

This study highlights the altered expression profiles associated with delayed diabetic wound healing, including reduced inflammation and collagen production. Further drug screening identified compound CG-930 as a novel therapeutic agent with significant potential to promote wound healing in diabetic conditions.

## Graphical Abstract

**Figure:**
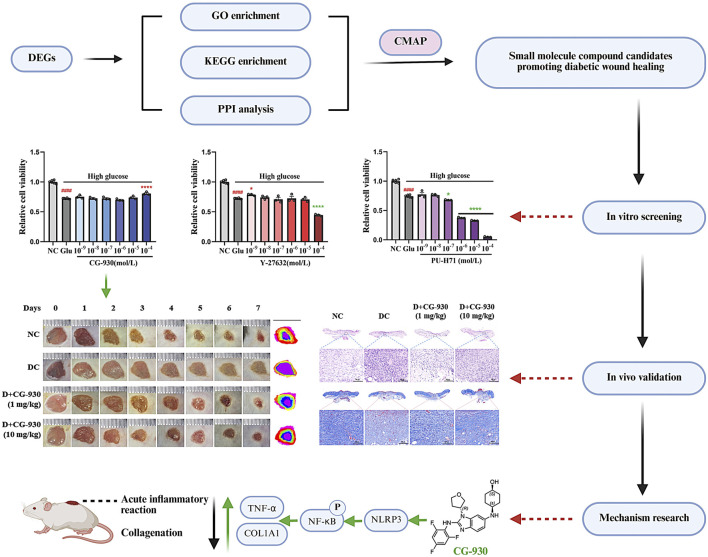
Created by Biorender.com.

## 1 Introduction

The global incidence of diabetes is steadily increasing, with approximately 20% of the patients potentially affected by diabetic wound (Patel, et al., 2019; GBD, 2021 Diabetes Collaborators, 2023). Among these, DFU caused by wounds are one of the most severe complications of diabetes, often leading to amputation and even endangering patients’ lives (Aldana and Khachemoune, 2020).

Normal wound healing follows a sequential cascade of events, including blood coagulation, inflammation, proliferation, and remodeling, which require precise coordination and interaction among multiple factors (Wallace, et al., 2024). However, in individuals with diabetes, adherence to this healing trajectory is often compromised, leading to stagnation at various stages (Dasari, et al., 2021). Impaired wound healing not only increases the risk of infection but also contributes to a range of complications (Park, et al., 2022).

Current clinical treatments for DFU include debridement, enhanced wound care, local offloading, and glycemic control (Chiu, et al., 2023; Gallagher, et al., 2024). Despite these approaches, effectively promoting the healing of chronic diabetic wounds remains a major challenge, with unsatisfactory cure rates (Burgess, et al., 2021). Globally, the number of approved drugs for promoting diabetic would healing is limited. Existing options include growth factor-based drugs (rhPDGF-BB/becaplermin, United States; rhEGF/Nepidermin, South Korea) (Yadav, et al., 2024) and natural product extracts (ON101, Taiwan; Curasite, United States) (Huang, et al., 2021; Chang, et al., 2024). Meanwhile, numerous candidate drugs are under preclinical or clinical investigation. However, significant obstacles persist in drug development. For instance, growth factor-based therapies demonstrate limited clinical efficacy (Corporation, 2012; Park, et al., 2014), and although ON101 has shown promising clinical outcomes (Huang, et al., 2021; Chang, et al., 2024), its mechanisms and characteristics require further elucidation (Huang, et al., 2021). Therefore, there is an urgent need to develop novel therapeutic agents to accelerate diabetic wound healing.

Recent advances have deepened our understanding of the molecular mechanisms underlying impaired diabetic wound healing, yet many gaps remain. Aberrant inflammatory responses are widely recognized as critical contributors to this process, characterized by complexity and dynamic changes (Matoori, et al., 2021). Early inflammation, in particular, plays a pivotal role in initiating the transition from the inflammatory to the proliferative phase (Dong, et al., 2020; Matoori, et al., 2021). However, the precise molecular events remain contentious. Some studies suggest that enhancing the acute inflammation may facilitate diabetic wound healing (Leal, et al., 2015; Matoori, et al., 2021), whereas others advocate suppressing pro-inflammatory responses in the initial wound phase to prevent chronic and excessive immune processes (Cai, et al., 2023; Zhao, et al., 2024). Notably, most omics analyses of diabetic wounds have focused on the late ulcerative stage of inflammation (Theocharidis, et al., 2022; Wang, et al., 2023). Identifying transcriptomic characteristics in the early inflammatory phase of diabetic wounds is therefore crucial for developing effective therapeutics.

This study aims to characterize the transcriptomic profile of early inflammatory phase in diabetic wound healing, identify DEGs, and leverage these findings to discover potential therapeutic candidates for enhancing diabetic wound repair. We employed bioinformatics to analyze the transcriptome of diabetic wounds in a humanized mice model and identified early inflammatory-phase transcriptomic features using the CTD database. Small-molecule compounds capable of reversing these expression profiles were subsequently identified via the CMap database. Then, the candidate compounds were validated by both *in vivo* and *in vitro* experiments, along with a detailed analysis of their mechanisms of action.

## 2 Materials and methods

### 2.1 Reagents

CG-930, Y-27632, NVP-AUY922, and PU-H71 were supplied by Med Chem Express Co., Ltd (Shanghai, China). RIPA, protease inhibitor, and phosphatase inhibitor were purchased from Solarbio (Beijing, China). The Cell Counting Kit-8 (CCK-8) was supplied by Meilunbio (Beijing, China). Streptozotocin (STZ) was obtained from Innochem (Beijing, China). Primary antibody NLRP3 (Cat. 15101T), p-JNK (Cat. 4668S), JNK (Cat. 3708S), p-NF-κB (Cat. 3033T), NF-κB (Cat. 8242T), COL1A1 (Cat. 72026T), α-actin (Cat. 19245S), and GAPDH (Cat. 2118S) were purchased from Cell Signaling Technology (Boston, United States). TNF-α (Cat. sc-52746) was supplied by Santa Cruz Biotechnology (California, United States). Goat Anti Rabbit IgG-HRP (Cat. P03S02L) and Goat Anti mouse IgG-HRP (Cat. P03S01M) were obtained from Gplink (Beijing, China).

### 2.2 Acquisition of differentially expressed genes

The target dataset GSE147890 was obtained from the GEO database at the National Center for Biotechnology Information (NCBI) (León, et al., 2020). This dataset comprises samples from seven normal mice and six diabetic mice, all of which were humanized, offering advantages for effective wound modeling and minimal individual variability. Tissue samples were collected for analysis at two time points: biopsies immediately after wound formation (0 h) and biopsies after 24 h post-injury (24 h). Total RNA was extracted from these samples for sequencing analysis, generating gene expression profiling data. Using GEO2R for online analysis (Barrett, et al., 2013), we identified DEGs between the wounds of diabetic and non-diabetic mice.

To enhance the reliability of data, we searched the CTD database (Davis, et al., 2023) using the keyword “diabetic foot ulcers” to retrieve a related gene dataset. We then identified overlapping genes between these two databases to obtain DEGs specifically associated with DFU.

### 2.3 GO and KEGG enrichment analyses

GO and KEGG analyses are widely used enrichment methods in bioinformatics (Chen, et al., 2017; Zhao, et al., 2019). In this study, we employed the ClusterProfiler package (Wu, et al., 2021) in R to conduct enrichment analyses on the DEGs. This approach allowed us to investigate the biological processes, cellular components, molecular functions, and signaling pathways associated with these genes, thereby providing preliminary insights into the factors linked to impaired wound healing in diabetes.

### 2.4 PPI analysis

In this study, we utilized the String database to conduct a protein-protein interaction (PPI) network analysis with the DEGs (Szklarczyk, et al., 2023). We set the reliability threshold for the analysis results to “high confidence” before importing the data into Cytoscape software (Doncheva, et al., 2019). “Degree” values for each protein’s interactions were calculated and the resultant visualizations were optimized accordingly. Finally, the CytoHubba plugin was employed to identify hub proteins associated with the disease (Chin, et al., 2014).

### 2.5 Identification of agent candidates based on CMap database

The CMap database is a widely used resource based on genome-wide transcriptional profiles that systematically characterizes various biological states, including diseases, physiological conditions, and drug responses (Subramanian, et al., 2017). In this study, we employed the “query” tool of the CMap database to input the DEGs. We selected the parameters “Gene expression (L1000), “Touchstone”, and “Individual Query”, which yielded small molecular compounds with opposing gene expression profile to the input genes and generated corresponding scoring values for each compound.

### 2.6 Cell culture

Fibroblast cell line (NIH-3T3) was obtained from Cell Resource Center (Cell), Institute of Basic Medical Sciences, Chinese Academy of Medical Sciences (Beijing China). NIH-3T3 fibroblasts were cultured in high-glucose DMEM supplemented with 10% FBS, 100 IU/mL penicillin, and 100 μg/mL streptomycin. To simulate high-glucose or high-lipid environments, the cells were treated with 40 mM exogenous glucose or 0.25 mM oleic acid for 24 h. Prior to adding the drug or modeling reagents, all FBS-containing media were replaced with FBS-free media. Cell viability was assessed using CCK-8 assay.

### 2.7 Animal modeling and drug treatments

All mice were acclimated for 1 week. After NC mice were randomly selected, the remaining mice were injected intraperitoneally with 120 mg/kg STZ. Fasting blood glucose levels were measured 1-week post-injection. Mice with fasting blood-glucose levels above 13.6 mmol/L were classified as T1D mice.

Following isoflurane anesthesia, the mice were depilated on their backs using depilation cream. To minimize continuous skin irritation, wound modeling was conducted 2 days post depilation. Four holes were created on the backs of the mice using an 8 mm diameter hole punch.

A ruler was placed above the wound for scale, and photographs was taken daily to assess the wound area, which was calculated using ImageJ software. The wound healing rate was determined as follows: Wound Healing Rate = [ (Wound area on day 0 - Wound area on day n)/Wound area on day 0] ×100%. Mice in treatment group received topical application of 1 and 10 mg/kg per day drugs for seven consecutive days, while mice in the NC group and DC group were treated with an equivalent volume of normal saline. On day 7 post-administration, we euthanized the mice under isoflurane anesthesia and collected tissue samples from the wounds for examination.

The animal study protocol was approved by the Ethics Committee of The Animal Care and Welfare Committee Institute of Materia Medica, CAMS&PUMC (protocol code 00004156, 2023.5.31).

### 2.8 Histological analysis

Wound tissues were fixed in 10% formalin. Transverse paraffin sections, 5 μm in thickness, were prepared and subjected to Hematoxylineosin (HE) and Masson staining. These staining methods were employed to evaluated the degree of inflammatory cell infiltration and collagen formation in the wound. These experiments were performed by Servicebio Co., Ltd.

### 2.9 Data and statistical analysis

Data are presented as means ± SEM. Statistical analysis was performed with GraphPad Prism 8.0 (GraphPad Software, Inc., CA, United States). ANOVAs were utilized for statistical comparisons as needed, with significance determined at P < 0.05.

## 3 Results

### 3.1 DEGs acquisition between normal and diabetic wounds from GEO database

The dataset GSE147890 was obtained from a diabetic skin humanized mice model in the GEO database (León, et al., 2020), and the GEO2R online tool was utilized to analyze DEGs between normal and diabetic wounds. The criteria for identifying DEGs included a p-value <0.05 and an absolute log_2_FoldChange >0.5.

**FIGURE 1:**
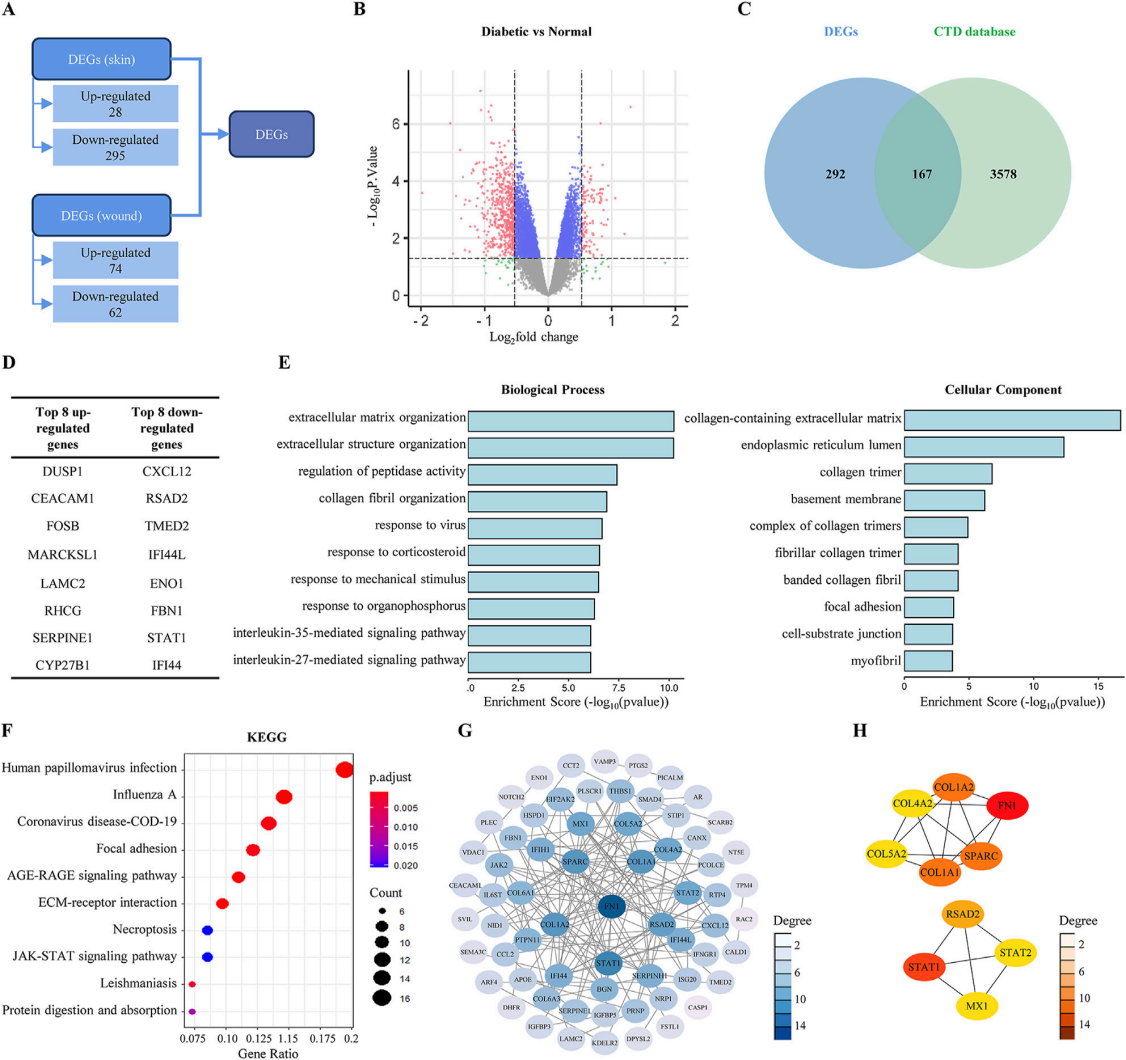
Predicting the compounds that promoting diabetic wound healing based on bioinformatics. **(A)** Screening for DEGs in the GSE147890 dataset. **(B)** Volcano plot of DEGs in diabetic skin/wound compared with normal skin/wound. **(C)** Venn diagram of DEGs in the GSE147890 dataset and genes associated with DFU disease in CTD database. **(D)** The top eight up- and downregulated genes. **(E)** GO enrichment results of DEGs. **(F)** KEGG enrichment results of DEGs. **(G)** PPI interaction network by Cytoscape. **(H)** The top 10 hub genes in the PPI screened by CytoHubba.

In the 2 mm tissue biopsy at 0 h, we identified a total of 323 DEGs in diabetic skin compared to normal skin, comprising 28 upregulated and 295 downregulated genes. In the 6 mm tissue biopsy at 24 h, a total of 136 DEGs were identified, with 74 upregulated and 62 downregulated genes. Combining the DEGs from both biopsies resulted 459 unique genes, including 102 upregulated and 357 downregulated genes (Figures 1A,B).

A search in the CTD using the keyword “diabetic foot ulcer” yielded 3,745 genes associated with DFU. Among the 459 DEGs identified, 167 genes overlapped with the CTD data (Figure 1C), including 18 upregulated genes (the top eight being *DUSP1*, *CEACAM1*, *FOSB*, *MARCKSL1*, *LAMC2*, *RHCG*, *SERPINE1*, and *CYP27B1*) and 149 downregulated genes (the top eight being *CXCL12*, *RSAD2*, *TMED2*, *IFI44L*, *ENO1*, *FBN1*, *STAT1*, and *IFI44*) (Figure 1D).

### 3.2 DEGs enrichment analyses results

GO and KEGG enrichment analyses of the 167 DEGs were performed using the ClusterProfiler package in R. The analyses indicated significantly enrichment in 786 biological processes, 72 cellular components, 61 molecular functions, and 44 signaling pathways (with counts >4). The GO enrichment results primarily pertained to the extracellular matrix and collagen, suggesting that drug design aimed at promoting diabetic wound healing could focus on these aspects (Figure 1E).

The KEGG analysis illustrated the enrichment of DEGs across multiple pathways, as illustrated in the bubble plot showing the ten pathways with the lowest p-values. The five most significant pathways associated with wound healing were focal adhesion, AGE-RAGE signaling pathway, ECM-receptor interaction, necroptosis, and the JAK-STAT signaling pathway (Figure 1F).

To further explore the interactions among the DEGs, we utilized the String database and identified ten Hub genes critical to the disease based on their scores: *FN1*, *STAT1*, *COL1A1*, *SPARC*, *COL1A2*, *RSAD2*, *MX1*, *STAT2*, *COL4A2*, and *COL5A2* (Figures 1G,H).

### 3.3 Identification of candidate compounds promoting diabetic wound healing by CMap

**FIGURE 2:**
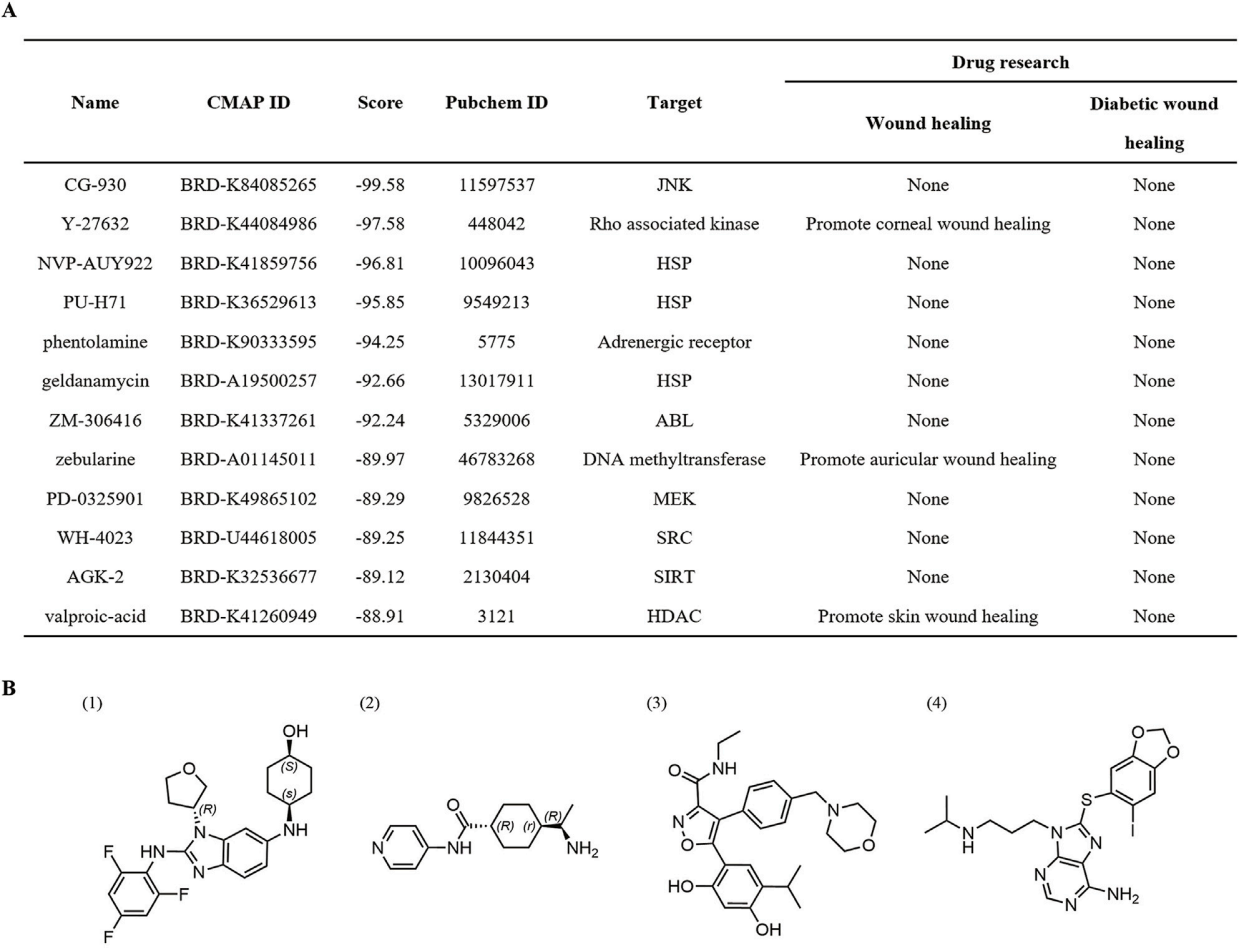
Screening for small molecule compounds based on CMap database. **(A)** Small molecule compounds screened by CMap. **(B)** The structure of (1) CG-930, (2) Y-27632, (3) NVP-AUY922, and (4) PU-H71.

Through comparative analysis of the 167 DEGs with the gene profile of existing small molecules in the CMap database, we identified 2,427 small molecule compounds that exhibited similar or opposing expression patterns to the DEGs. The scores of these compounds ranged from - 99.58 to +99.58, with 12 small molecules scored below - 80 (Figure 2A).

Among them, the Rho-associated kinase inhibitor Y-27632 has been reported to accelerate endothelial cell regeneration and promote corneal wound healing (Okumura, et al., 2015; Sun, et al., 2015; Zhang, et al., 2021). Phentolamine, a reversible adrenergic receptor antagonist, has not been previously studied for its effect on wound healing; however, research suggests that β-adrenergic receptor blockers can enhance diabetic wound healing through various mechanisms, including accelerating re-epithelialization, promoting angiogenesis, alleviating neuropathy, and modulating inflammatory responses and growth factors (Sun, et al., 2020; Jia, et al., 2024). The DNA methyltransferase inhibitor zebularine promoted the healing of auricular wounds by inducing the expression of genes associated with regenerative responses and modifying cellular behavior (Sass, et al., 2019). Additionally, valproic acid was reported to facilitate skin wound healing through mechanisms that reduce inflammatory responses, enhance the clearance of apoptotic cells, and increase keratinocyte viability (Lee, et al., 2012; Chen, et al., 2023). These studies further validate the reliability of our screening model.

In this study, we selected top four compounds based the CMap scores for further investigation at the cellular level. The selected compounds include CG-930, Y-27632, NVP-AUY922, and PU-H71. (Figure 2B).

### 3.4 *In vitro* assessment of compounds activities

Fibroblasts, which are mesoderm-derived cells commonly found in connective tissue, play a crucial role at multiple stages of wound healing including inflammation (Ekchariyawat, et al., 2015), proliferation (Bainbridge, 2013; Wang, et al., 2018), and remodeling (Talbott, et al., 2022). Fibroblasts are often used to evaluate the effects of wound healing drugs (Shen, et al., 2020; Latif, et al., 2024). Therefore, we selected fibroblasts as experimental subjects to evaluate the effects of compounds on the cell viability under normal, high-glucose, and high-lipid conditions.

**FIGURE 3:**
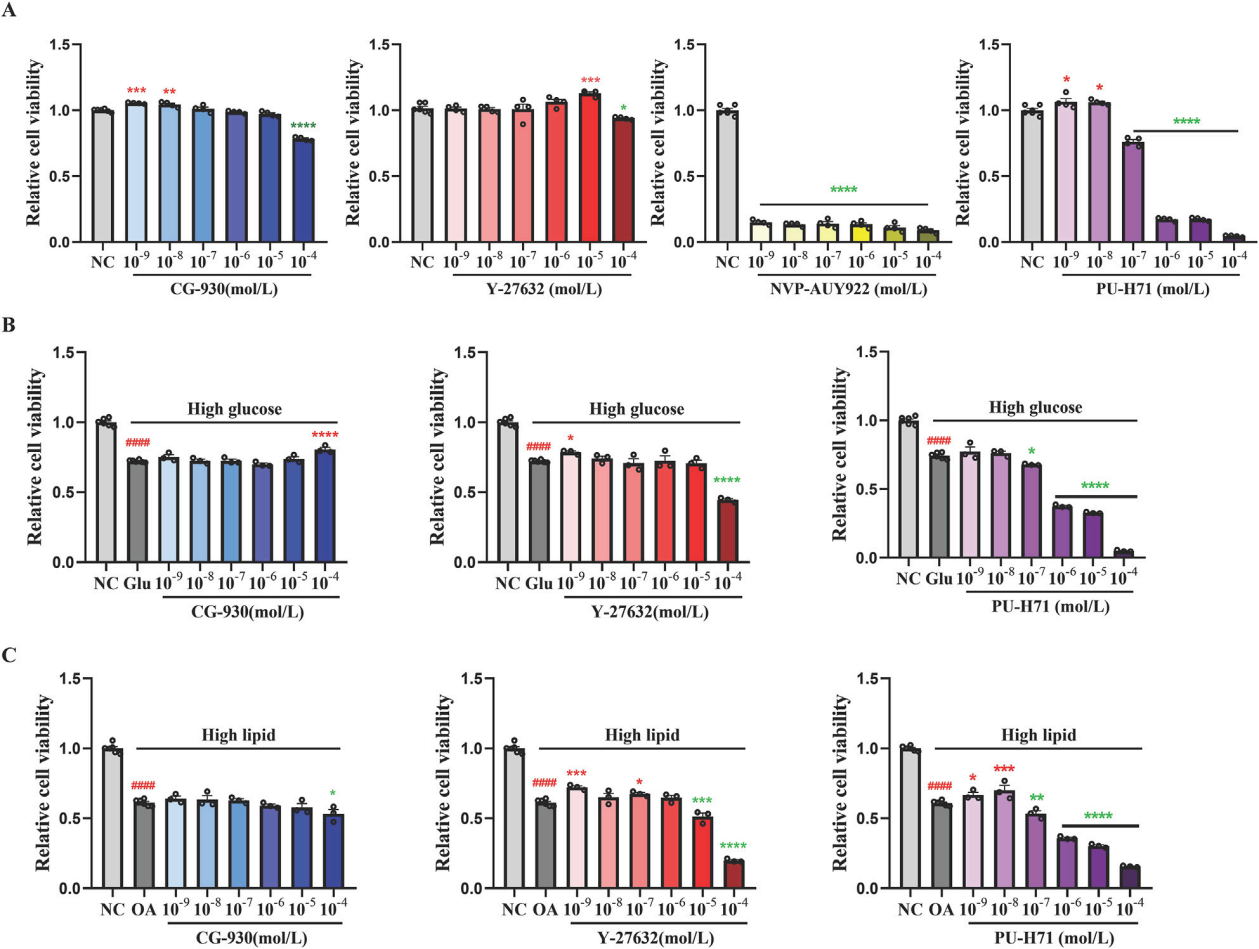
Evaluating the compounds that promoting proliferation of fibroblasts. **(A)** The effects of compounds on the proliferative activity of normal fibroblasts. **(B)** The effects of compounds on the proliferative activity of high glucose-induced fibroblasts. **(C)** The effects of compounds on the proliferative activity of high lipid-induced fibroblasts. Data presented are individual values with means ± SEM from n = 3-6 for each group. Statistical analysis tested by one-way ANOVA. ####*p* < 0.0001 vs the NC group. *p < 0.05, **p < 0.01, ***p < 0.001, and ****p < 0.0001 vs the model group.

In the normal fibroblast model, CG-930 (10^–9^ and 10^–8^ mol/L), Y-27632 (10^–5^ mol/L), and PU-H71 (10^–9^ and 10^–8^ mol/L) exhibited proliferative activity. While NVP-AUY922 demonstrated significant toxicity (Figure 3A), leading us to exclude this compound from subsequent studies. In the high-glucose-induced fibroblast model, 40 mM glucose significantly inhibited fibroblast proliferation, while CG-930 (10^–4^ mol/L) and Y-27632 (10^–9^ mol/L) ameliorated this inhibitory effect (Figure 3B). In the high-lipid-induced fibroblast model, Y-27632 (10^–9^ and 10^–7^ mol/L) and PU-H71 (10^–9^ and 10^–8^ mol/L) significantly reversed the inhibitory effects of high lipid on fibroblast proliferation (Figure 3C).

Based on the results from these three models, although PU-H71 demonstrated a favorable ability to promote fibroblast proliferation, its narrow therapeutic window precluded further investigation. Ultimately, CG-930 and Y-27632 were selected for evaluating their effects in the type 1 diabetes (T1D) mice wound healing model.

### 3.5 Effect of CG-930 on diabetic wound healing in T1D mice

**FIGURE 4:**
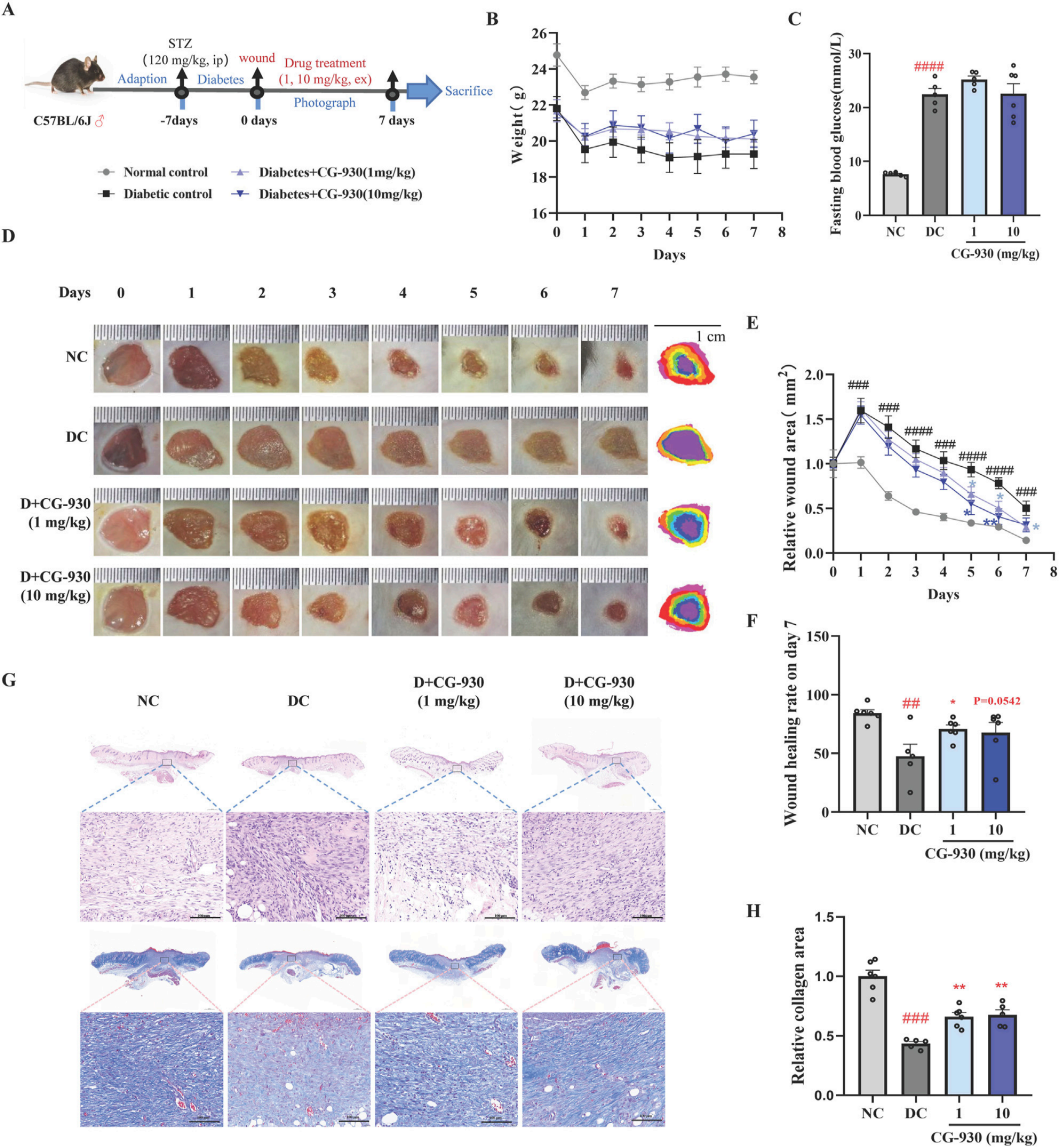
Validating the compounds that promoting diabetic wound healing in the mice. **(A)** A schematic diagram of experimental process. **(B)** The effect of CG-930 on the body weight. **(C)** Fasting blood glucose on day 7. **(D)** Representative images of wounds. **(E)** Relative wound area on day 0 to day 7. **(F)** Wound healing rate on day 7. **(G)** HE stain and Masson stain on the day 7. **(H)** Relative collagen area analysed by Masson staining on day 7. Data presented are individual values with means ± SEM from n = 6 for each group. Statistical analysis tested by one-way ANOVA and two-way ANOVA (figure B and E). ##*p* < 0.01 and ####*p* < 0.0001 vs the NC group. *p < 0.05 vs the DC group.

In this study, compared to the normal control (NC) group, the body weights of the T1D mice were significantly reduced. Topical administration of CG-930 and Y-27632 (1 and 10 mg/kg) did not significantly influence the body weight of T1D mice, although CG-930 demonstrated a tendency to promote weight gain (Figures 4A,B, Supplementary Figure S1A). Fasting blood glucose levels were significantly elevated in T1D mice; however, no significant effects were observed following treatment with either compound (Figure 4C, Supplementary Figure S1B).

We photographed the wounds on the backs of the mice daily during the treatment and measured their areas. The results indicated that 1 mg/kg of CG-930 significantly reduced the wound area of T1D mice on days 5, 6, and 7. Similarly, 10 mg/kg of CG-930 reduced the wound area on days 5, and 6 post treatment. In normal mice, the wound reached 84% closure by day 7, whereas the T1D group exhibited a significantly lower closure of only 48%. Following treatment with 1 and 10 mg/kg of CG-930, the closure increased to 71% and 68%, respectively (Figures 4D–F). In contrast, Y-27632 did not demonstrate significant effects on diabetic wound healing (Supplementary Figure S1C).

HE and Masson staining results indicated that the diabetic control (DC) group exhibited more pronounced infiltration of inflammatory cells and impaired collagen production at the wound site compared to the NC group. In contrast, the CG930-treated group showed a significant reduction in inflammation, especially at the 1 mg/kg dosage. Furthermore, both the 1 mg/kg and 10 mg/kg doses of CG-930 significantly enhanced collagen production (Figures 4G,H).

### 3.6 CG-930 promotes early wound inflammation and collagen formation

**FIGURE 5:**
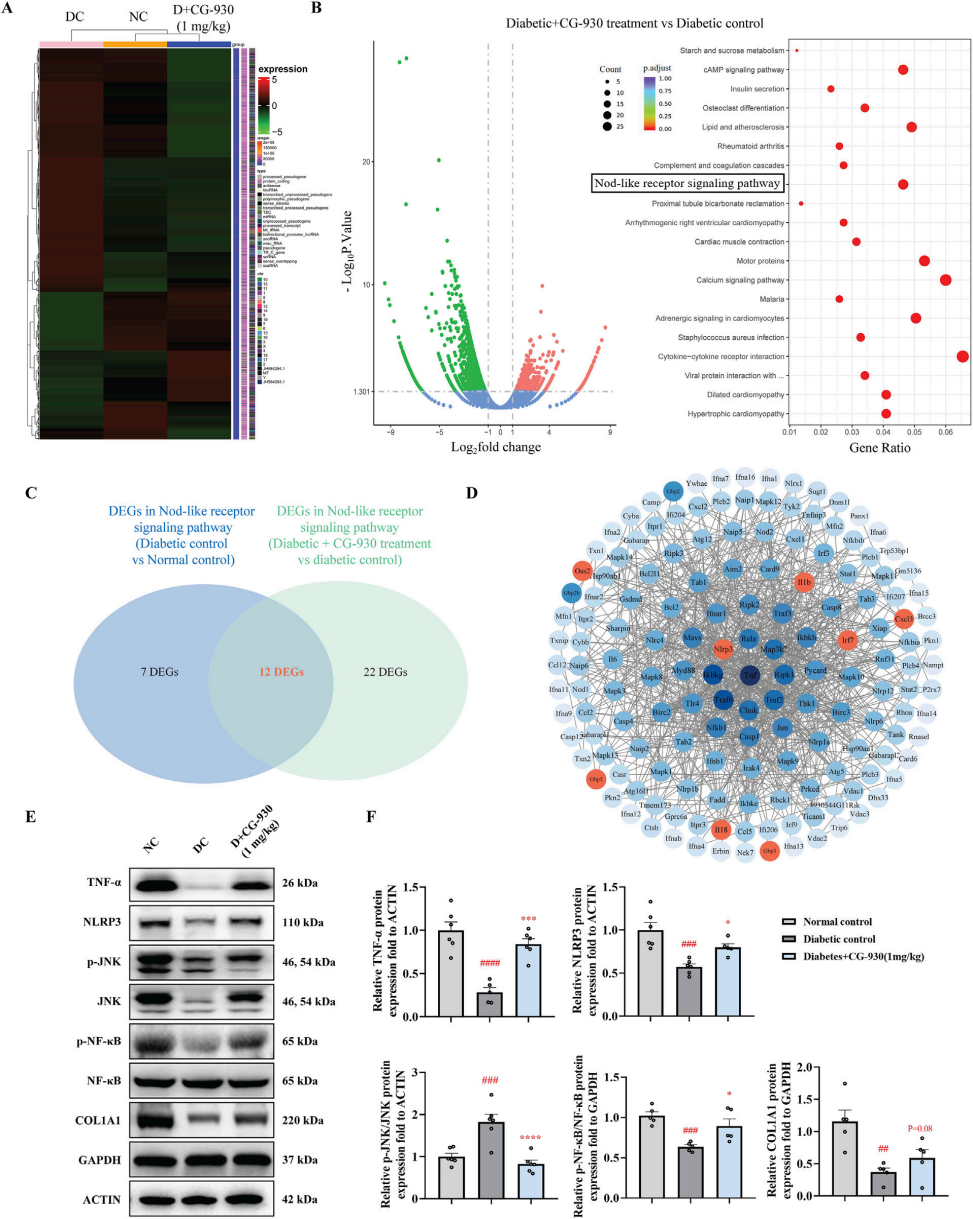
Study on mechanism of CG-930 promoting wound healing in T1D mice. **(A)** Correlation heat map of mRNA expression in wounds of NC, DC, and CG-930 (1 mg/kg) treatment groups at 24 h. **(B)** Volcano plot and KEGG dotplot of DEGs. **(C)** Venn diagram of DEGs in NLR signaling pathway between DC vs NC and CG-930 treatment vs DC. **(D)** PPI network map of all genes in the NLR signaling pathway. **(E)** Representative bands of Western blot. **(F)** Immunoblot analyses of TNF-α, NLRP3, p-JNK/JNK, p-NF-κB/NF-κB, and COL1A1. Data presented are individual values with means ± SEM from n = 5-6 for each group. Statistical analysis tested by one-way ANOVA. ##*p* < 0.01, ###*p* < 0.001, and ####*p* < 0.0001 vs the NC group. *p < 0.05, ***p < 0.001, and ****p < 0.0001 vs the DC group.

RNA sequencing was performed (Beijing novogene Co., Ltd.) on wound tissue collected 24 h after injury to preliminarily assess the mechanisms. The correlation in RNA expression between the NC and CG-930-treated groups was higher than that between the NC and DC groups (Figure 5A), suggesting that CG-930 treatment aligns RNA expression more closely with normal levels. We analyzed the expression of genes related to collagen synthesis, inflammation, and growth factors from RNA sequencing results (SF. 2A), demonstrating that CG-930 treatment enhanced the expression of these genes, including hub genes identified in prior PPI analysis. We analyzed the DEGs between the DC group and the NC group, as well as between the CG-930 treatment group and the DC group. The results indicated that both two groups of DEGs were significantly enriched in the NLR signaling pathway (Figure 5B, Supplementary Figure S2B), which is strongly associated with inflammation (Liu, et al., 2019; Platnich and Muruve, 2019). Further analysis of DEGs in these two groups within NLR signaling pathway revealed 12 overlapping genes: *Cxcl3*, *Il1b*, *Nlrp3*, *Defb6*, *Oas3*, *Gbp3*, *Irf7*, *Gbp5*, *Mefv*, *Il18*, *Gpsm3*, and *Oas2* (Figure 5C). Among them, eight genes were highlighted in the PPI network map of all genes involved in the NLR signaling pathway (Figure 5D); four were excluded due to weak connections with other genes. NLRP3 exhibited closer interactions with other proteins, leading us to select it and its related proteins for subsequent validation.

We observed a significant decrease in the expressions of TNF-α, NLRP3, p-NF-κB/NF-κB, and COL1A1 in the model group. However, CG-930 significantly upregulated these gene expressions, thereby promoting early-stage inflammation and collagen formation, which facilitating the wound healing process (Figures 5E,F).

## 4 Discussion

This study employed informatics methods and integrated *in vitro* and *in vivo* validation to discover that the small molecule compound CG-930 plays a novel role in promoting diabetic wound healing. In this study, we used the GEO dataset of humanized diabetic mice wounds, and combined it with genes associated with diabetic wounds in the CTD database to obtain disease associated DEGs. After *in vitro* screening, we validated two promising compounds. Then, we found that compound CG-930 can enhance diabetic mice wound healing. Mechanistic exploration indicated that CG-930s promotion of diabetic wound healing may be linked to enhanced early inflammation and increased collagen production.

Currently, there are no reports on the identification of drugs that promote wound healing through CMap. However, CMap technology has been previously applied in drug discovery (Subramanian, et al., 2017). This approach assumes that the cellular signatures reflect pharmacologic perturbagens. If small molecules have cellular signatures opposite to those of a disease may have therapeutic effects (Subramanian, et al., 2017), and numerous drug discovery outcomes have been reported (Zhao, et al., 2023). In fact, the expression of multiple genes (including inflammatory factor-related genes) in diabetic skin is generally high, and the acute inflammatory response typically observed in normal wounds is not effectively induced after wound stimulation in diabetic conditions (Matoori, et al., 2021). Additionally, in STZ-induced type 1 diabetic mice, we observed that the expression levels of most *Fgfs* in diabetic wounds were significantly lower than in normal wounds (Wang, et al., 2023). This complex alteration in gene expression profile caused by basal and wound may make it difficult to use CMap to discover drugs to promote wound healing.

The study of diabetic wound healing in patients is limited mainly by technical and ethical considerations as well as by the complexity and heterogeneity of the disease (León, et al., 2020). The establishment of immunocompromised mouse models engrafted with human skin equivalents offers an innovative solution for simulating human cutaneous physiology and pathology (Agarwal, et al., 2020). Skin humanized mice display reduced wound contraction, a slower phenotypic transition from M1 macrophages to M2 macrophages, and delayed granulation tissue formation with poor vascular and cellular density, resluting its wound healing profile is more analogous to that of human wound (Escámez, et al., 2008; Martínez-Santamaría, et al., 2013; Zomer and Trentin, 2018; León, et al., 2020). The drug discovery strategy employed in this project utilized skin humanized mice to minimize species differences and better control the sampling times compared to human tissues. We combined skin and wound DEGs with diabetic foot ulcer-related genes from the CTD database to identify more specific cellular signatures for drug discovery.

We found that CG-930 can enhance the acute inflammatory response of diabetic wounds and accelerate wound healing. Typically, wounds remain in the inflammatory phase for approximately 24 h post-injury (Landén, et al., 2016). However, in diabetic conditions, inflammation reduces in the initial stage and subsequently increases (Matoori, et al., 2021; Sharifiaghdam, et al., 2022; Worsley, et al., 2023). This ultimately triggers excessive and persistent inflammation (Matoori, et al., 2021). Current, drug discovery efforts targeting chronic wound treatment primarily focus on anti-inflammatory strategies (Cai, et al., 2023). In our study, we observed a decrease in the early inflammatory response of diabetic wounds, and treatment with CG-930 significantly reversed this change. Promoting inflammation during the acute phase of diabetic wounds has been shown to prevent the transition to chronic wounds (Matoori, et al., 2021). Meanwhile, our findings revealed that all ten Hub genes from GSE147890 were downregulated, indicating that under diabetic conditions, the early inflammatory response is compromised, impeding subsequent later collagen synthesis and extracellular matrix formation. For instance, *STAT1* and *STAT2* function as signal transducers and transcription factor activators linked to inflammation (Owen, et al., 2019; Agashe, et al., 2022); *RSAD2* is an interferon-related DNase that plays a pivotal role in immune regulation (Chen, et al., 2024); *MX1*, known for inhibiting viral entry (Haller, et al., 2015), may also serve as a potential inflammatory target in diabetes (Wang, et al., 2024). Similarly, *FN1* is a component of the temporary matrix (Steffens, et al., 2012) and plays a role in wound healing (Gimeno-LLuch, et al., 2022); *COL1A1*, *COL1A2*, *COL4A2* and *COL5A2* are members of the collagen family (Peng, et al., 2022); while *SPARC* is a matrix cell protein (Brekken and Sage, 2001), the overexpression of which can promote wound healing (Wu, et al., 2024).

Our results suggest that CG-930 may activate the NLR signaling pathway, promoting early inflammatory response and the expression of growth factor, which in turn facilitates fibroblast proliferation and collagen accumulation, thereby enhancing wound healing. Inflammatory factors, growth factors, and inflammatory cells such as macrophages, can stimulate fibroblast proliferation (Walton, et al., 2017). The proliferation and maintenance of fibroblast function are crucial in diabetic wounds healing, aiding the transition from the pro-inflammatory stage to the anti-inflammatory stage (Liu, et al., 2022). NLRs are vital component of the mammalian innate immune system, and regulate inflammation through inflammasomes, NF-κB, MAPK, IL-1β, etc. Notably, some studies indicate that inhibiting NLRP3 inflammasome-induced inflammation resolution can enhance granulation tissue formation and accelerate re-epithelialization and wound closure (Cavalcante-Silva and Koh, 2023). This discrepancy may arise from variations sampling time post-modeling.

CG-930 has been previously reported as a c-Jun N-terminal kinase (JNK) inhibitor (Subramanian, et al., 2017). JNK is involved in various physiological processes (Yung and Giacca, 2020). STZ was reported could induce increased phosphorylation levels of JNK in wounds (Zhou, et al., 2021), yet research on JNK expression in diabetic wounds is lacking. Our findings indicate that TNF-ɑ expression is significantly reduced in diabetic wounds, while TNF-α is known to activate JNK (Natoli, et al., 1997). The expression of JNK in diabetic mice in this study was also significantly reduced; however, CG-930 treatment led to an increase in JNK within wounds. Thus, we posit that its healing-promoting effect is not mediated through JNK inhibition, which may clarify the limited efficacy of certain anti-inflammatory drugs on wound healing.

There are several issues remain to be addressed in this study. First, the mechanisms by which CG-930 regulates gene expression profiles require further investigation, particularly regarding on inflammatory gene expression. Second, the evaluation of treating which wound phase has the better efficacy for CG-930.

In summary, this study establishes a drug discovery strategy for enhancing diabetic wound healing based on informatics drug screening, complemented by *in vivo* and *in vitro* evaluations. We determine that CG-930 promotes wound healing in STZ-induced diabetic mice and suggest its potential use in the early stages of diabetic wounds or following debridement, providing a reference for early treatment strategies in diabetic wound management.

## Data Availability

The raw sequence data reported in this paper have been deposited in the Genome Sequence Archive (Genomics, Proteomics and Bioinformatics 2021) in National Genomics Data Center (Nucleic Acids Res 2022), China National Center for Bioinformation/Beijing Institute of Genomics, Chinese Academy of Sciences (GSA: CRA022844) that are publicly accessible at https://ngdc.cncb.ac.cn/gsa.
