# Supplementary material for: Identification of compounds to promote diabetic wound healing based on transcriptome signature
Source: Front Pharmacol. 2025 Jun 2;16:1576056. doi: 10.3389/fphar.2025.1576056 (PMC12171139; doi:10.3389/fphar.2025.1576056)
Supplement: Supplementary file 1 [file DataSheet1.zip › Supplementary material/1. Supplementary_Material.docx]

Supplementary Material

# Supplementary Figures and Tables

## Supplementary Table

Table S1. 167 DEGs in the GSE147890 dataset which associated with DFU disease in CTD database

| **ID** | **Gene.symbol** | **P.Value** | **adj.P.Val** | **logFC** | **Gene.title** |
| --- | --- | --- | --- | --- | --- |
| 201041_s_at | DUSP1 | 2.53E-07 | 0.00281 | 1.29497439 | Dual specificity phosphatase 1 |
| 209498_at | CEACAM1 | 3.95E-04 | 0.06421 | 1.05466869 | Carcinoembryonic antigen related cell adhesion molecule 1 |
| 202768_at | FOSB | 1.04E-03 | 0.09005 | 0.86749738 | FosB proto-oncogene, AP-1 transcription factor subunit |
| 200644_at | MARCKSL1 | 2.73E-05 | 0.03165 | 0.85866194 | MARCKS like 1 |
| 207517_at | LAMC2 | 1.87E-04 | 0.05479 | 0.85777324 | laminin subunit gamma 2 |
| 219554_at | RHCG | 5.48E-04 | 0.07268 | 0.80842052 | Rh family C glycoprotein |
| 202627_s_at | SERPINE1 | 3.91E-04 | 0.06413 | 0.71737057 | Serpin family E member 1 |
| 205676_at | CYP27B1 | 5.35E-04 | 0.07255 | 0.71065906 | Cytochrome P450 family 27 subfamily B member 1 |
| 211889_x_at | CEACAM2 | 3.10E-04 | 0.06021 | 0.62968802 | Carcinoembryonic antigen related cell adhesion molecule 2 |
| 211610_at | KLF6 | 4.79E-05 | 0.0415 | 0.61954068 | Kruppel like factor 6 |
| 203382_s_at | APOE | 1.93E-03 | 0.07096 | 0.57713573 | Apolipoprotein E |
| 213603_s_at | RAC2 | 5.56E-04 | 0.07285 | 0.5761281 | Ras-related C3 botulinum toxin substrate 2 (rho family, small GTP binding protein Rac2) |
| 204698_at | ISG20 | 4.25E-04 | 0.06628 | 0.57219858 | Interferon stimulated exonuclease gene 20 |
| 202295_s_at | CTSH | 2.34E-04 | 0.03166 | 0.56703525 | Cathepsin H |
| 220016_at | AHNAK | 1.43E-04 | 0.02849 | 0.54472861 | AHNAK nucleoprotein |
| 208961_s_at | KLF6 | 1.86E-04 | 0.05479 | 0.54414167 | Kruppel like factor 6 |
| 222348_at | MAST4 | 3.36E-04 | 0.03527 | 0.53966336 | Microtubule associated serine/threonine kinase family member 4 |
| 204948_s_at | FST | 2.48E-04 | 0.05607 | 0.50633272 | Follistatin |
| 212091_s_at | COL6A1 | 2.87E-04 | 0.03265 | -0.50177 | Collagen type VI alpha 1 chain |
| 212930_at | ATP2B1 | 1.88E-03 | 0.07037 | -0.50396374 | ATPase plasma membrane Ca2+ transporting 1 |
| 211367_s_at | CASP1 | 3.56E-03 | 0.09609 | -0.50426771 | Caspase 1 |
| 209344_at | TPM4 | 8.25E-04 | 0.04735 | -0.51056862 | Tropomyosin 4 |
| 210299_s_at | FHL1 | 1.18E-03 | 0.05544 | -0.5113587 | Four and a half LIM domains 1 |
| 209708_at | MOXD1 | 3.25E-03 | 0.09222 | -0.51232287 | Monooxygenase DBH like 1 |
| 201792_at | AEBP1 | 3.09E-03 | 0.08973 | -0.51404964 | AE binding protein 1 |
| 207480_s_at | MEIS2 | 3.61E-04 | 0.06192 | -0.51925112 | Meis homeobox 2 |
| 201657_at | ARL1 | 2.53E-06 | 0.00941 | -0.51942244 | ADP ribosylation factor like GTPase 1 |
| 202566_s_at | SVIL | 1.49E-04 | 0.02898 | -0.51985272 | Supervillin |
| 200008_s_at | GDI2 | 7.88E-04 | 0.04668 | -0.52011509 | GDP dissociation inhibitor 2 |
| 202862_at | FAH | 1.52E-03 | 0.06229 | -0.5210077 | Fumarylacetoacetate hydrolase |
| 208454_s_at | CPQ | 2.03E-03 | 0.07245 | -0.52347836 | Carboxypeptidase Q |
| 222201_s_at | CASP8AP2 | 6.66E-04 | 0.07732 | -0.52366752 | Caspase 8 associated protein 2 |
| AFFX-HUMISGF3A/M97935_MA_at | STAT1 | 1.21E-03 | 0.05592 | -0.52804229 | Signal transducer and activator of transcription 1 |
| 200806_s_at | HSPD1 | 2.01E-03 | 0.07221 | -0.52992424 | Heat shock protein family D (Hsp60) member 1 |
| 201150_s_at | TIMP3 | 7.49E-04 | 0.04584 | -0.53212862 | TIMP metallopeptidase inhibitor 3 |
| 202784_s_at | NNT | 4.11E-04 | 0.03778 | -0.54254918 | Nicotinamide nucleotide transhydrogenase |
| 217140_s_at | VDAC1 | 7.93E-04 | 0.04674 | -0.54600444 | Voltage dependent anion channel 1 |
| AFFX-HSAC07/X00351_M_at | ACTB | 3.59E-03 | 0.09609 | -0.54696991 | Actin beta |
| 203939_at | NT5E | 1.98E-03 | 0.07146 | -0.54776704 | 5'-nucleotidase ecto |
| 203060_s_at | PAPSS2 | 1.53E-03 | 0.06232 | -0.54907653 | 3'-phosphoadenosine 5'-phosphosulfate synthase 2 |
| 205842_s_at | JAK2 | 1.35E-04 | 0.02802 | -0.55558591 | Janus kinase 2 |
| 201259_s_at | SYPL1 | 6.48E-05 | 0.01933 | -0.55605329 | Synaptophysin like 1 |
| 202092_s_at | ARL2BP | 1.18E-04 | 0.02729 | -0.55776807 | ADP ribosylation factor like GTPase 2 binding protein |
| 205170_at | STAT2 | 2.73E-03 | 0.08535 | -0.55932383 | Signal transducer and activator of transcription 2 |
| 222040_at | HNRNPA1 | 1.10E-03 | 0.09196 | -0.56057992 | Heterogeneous nuclear ribonucleoprotein A1 |
| 202403_s_at | COL1A2 | 2.59E-05 | 0.01884 | -0.56176469 | Collagen type I alpha 2 chain |
| 211110_s_at | AR | 1.02E-03 | 0.08937 | -0.56370364 | Androgen receptor |
| 203789_s_at | SEMA3C | 2.84E-04 | 0.03265 | -0.56983334 | Semaphorin 3C |
| 203357_s_at | CAPN7 | 1.86E-04 | 0.0302 | -0.57020299 | Calpain 7 |
| 201091_s_at | CBX3 | 1.98E-04 | 0.03063 | -0.5713824 | Chromobox 3 |
| 201664_at | SMC4 | 1.27E-03 | 0.09697 | -0.57147216 | Structural maintenance of chromosomes 4 |
| 218162_at | OLFML3 | 1.72E-03 | 0.06701 | -0.57372167 | Olfactomedin like 3 |
| 218656_s_at | LHFP | 1.26E-03 | 0.05675 | -0.57655265 | Lipoma HMGIC fusion partner |
| 201063_at | RCN1 | 1.03E-03 | 0.05281 | -0.57666795 | Reticulocalbin 1 |
| 213429_at | BICC1 | 1.27E-03 | 0.05682 | -0.57761108 | BicC family RNA binding protein 1 |
| 211749_s_at | VAMP3 | 8.73E-04 | 0.04921 | -0.57800708 | Vesicle associated membrane protein 3 |
| 201262_s_at | BGN | 5.32E-04 | 0.04066 | -0.57892271 | Biglycan |
| 211719_x_at | FN1 | 3.11E-04 | 0.03364 | -0.58609634 | Fibronectin 1 |
| 203813_s_at | SLIT3 | 5.63E-04 | 0.04128 | -0.58733046 | Slit guidance ligand 3 |
| 212196_at | IL6ST | 3.57E-04 | 0.03632 | -0.58806364 | Interleukin 6 signal transducer |
| 201560_at | CLIC4 | 2.57E-04 | 0.03216 | -0.58821141 | Chloride intracellular channel 4 |
| 213294_at | EIF2AK2 | 1.29E-03 | 0.05703 | -0.5899492 | Eukaryotic translation initiation factor 2 alpha kinase 2 |
| 207828_s_at | CENPF | 4.60E-04 | 0.06737 | -0.59002401 | Centromere protein F |
| 203961_at | NEBL | 1.05E-03 | 0.09005 | -0.59218047 | Nebulette |
| 201438_at | COL6A3 | 9.31E-04 | 0.05024 | -0.59260505 | Collagen type VI alpha 3 chain |
| 210495_x_at | FN1 | 3.74E-04 | 0.0369 | -0.59329225 | Fibronectin 1 |
| 202310_s_at | COL1A1 | 2.88E-05 | 0.01884 | -0.59899456 | Collagen type I alpha 1 chain |
| 203196_at | ABCC4 | 3.16E-04 | 0.06021 | -0.60057119 | ATP binding cassette subfamily C member 4 |
| 204748_at | PTGS2 | 1.23E-04 | 0.02757 | -0.60168181 | Prostaglandin-endoperoxide synthase 2 |
| 201337_s_at | VAMP3 | 3.89E-04 | 0.03737 | -0.60609002 | Vesicle associated membrane protein 3 |
| 209969_s_at | STAT1 | 6.94E-04 | 0.07779 | -0.60671607 | Signal transducer and activator of transcription 1 |
| 202817_s_at | SS18 | 6.34E-05 | 0.01933 | -0.60738433 | SS18, nBAF chromatin remodeling complex subunit |
| 210298_x_at | FHL1 | 2.81E-03 | 0.08681 | -0.60742692 | Four and a half LIM domains 1 |
| 200665_s_at | SPARC | 1.09E-04 | 0.02666 | -0.61072797 | Secreted protein acidic and cysteine rich |
| 211966_at | COL4A2 | 2.96E-03 | 0.08827 | -0.61160331 | Collagen type IV alpha 2 chain |
| 200698_at | KDELR2 | 1.84E-04 | 0.0302 | -0.61626206 | KDEL endoplasmic reticulum protein retention receptor 2 |
| 202527_s_at | SMAD4 | 6.04E-05 | 0.01924 | -0.61684819 | SMAD family member 4 |
| 212464_s_at | FN1 | 1.65E-04 | 0.02898 | -0.61773235 | Fibronectin 1 |
| 216442_x_at | FN2 | 4.58E-04 | 0.03805 | -0.61847073 | Fibronectin 2 |
| 201946_s_at | CCT2 | 1.50E-04 | 0.02898 | -0.62269259 | Chaperonin containing TCP1 subunit 2 |
| 205542_at | STEAP1 | 4.49E-04 | 0.03805 | -0.62356049 | Six transmembrane epithelial antigen of the prostate 1 |
| 201663_s_at | SMC4 | 9.97E-04 | 0.05199 | -0.62525001 | Structural maintenance of chromosomes 4 |
| 209337_at | PSIP1 | 2.19E-04 | 0.05479 | -0.62843046 | PC4 and SFRS1 interacting protein 1 |
| 204240_s_at | SMC2 | 2.22E-04 | 0.05496 | -0.62954587 | Structural maintenance of chromosomes 2 |
| 208747_s_at | C1S | 3.24E-04 | 0.03472 | -0.63324432 | Complement component 1, s subcomponent |
| 202430_s_at | PLSCR1 | 6.19E-04 | 0.04206 | -0.63454586 | Phospholipid scramblase 1 |
| 204163_at | EMILIN1 | 2.52E-03 | 0.08243 | -0.63464988 | Elastin microfibril interfacer 1 |
| 221139_s_at | CSAD | 1.06E-04 | 0.04818 | -0.63687156 | Cysteine sulfinic acid decarboxylase |
| 212298_at | NRP1 | 2.47E-04 | 0.03215 | -0.64167496 | Neuropilin 1 |
| 208782_at | FSTL1 | 2.40E-04 | 0.03166 | -0.64744541 | Follistatin like 1 |
| 201096_s_at | ARF4 | 6.83E-04 | 0.0441 | -0.64885883 | ADP ribosylation factor 4 |
| 209356_x_at | EFEMP2 | 2.86E-03 | 0.08736 | -0.65300722 | EGF containing fibulin like extracellular matrix protein 2 |
| 202766_s_at | FBN1 | 2.65E-04 | 0.0323 | -0.65363621 | Fibrillin 1 |
| 210284_s_at | TAB2 | 8.78E-04 | 0.04932 | -0.65510081 | TGF-beta activated kinase 1/MAP3K7 binding protein 2 |
| 221729_at | COL5A2 | 2.48E-05 | 0.01884 | -0.65734355 | Collagen type V alpha 2 chain |
| 200762_at | DPYSL2 | 1.57E-04 | 0.02898 | -0.66135834 | Dihydropyrimidinase like 2 |
| 39249_at | AQP3 | 4.65E-04 | 0.03823 | -0.66794429 | Aquaporin 3 (Gill blood group) |
| 204822_at | TTK | 1.51E-04 | 0.05454 | -0.67445034 | TTK protein kinase |
| 210839_s_at | ENPP2 | 2.42E-03 | 0.08033 | -0.67796618 | Ectonucleotide pyrophosphatase/phosphodiesterase 2 |
| 201539_s_at | FHL1 | 2.96E-03 | 0.08827 | -0.67862198 | Four and a half LIM domains 1 |
| 201646_at | SCARB2 | 1.17E-03 | 0.05522 | -0.68345672 | Scavenger receptor class B member 2 |
| 208867_s_at | CSNK1A1 | 1.94E-04 | 0.03063 | -0.68830215 | Casein kinase 1 alpha 1 |
| 208852_s_at | CANX | 7.74E-05 | 0.02104 | -0.68901593 | Calnexin |
| 202965_s_at | CAPN6 | 2.25E-03 | 0.07681 | -0.69149974 | Calpain 6 |
| 221729_at | COL5A2 | 1.19E-03 | 0.09548 | -0.69964592 | Collagen type V alpha 2 chain |
| 213226_at | CCNA2 | 8.23E-05 | 0.04447 | -0.70328588 | Cyclin A2 |
| 211676_s_at | IFNGR1 | 1.25E-04 | 0.02767 | -0.7042755 | Interferon gamma receptor 1 |
| 214505_s_at | FHL1 | 8.67E-04 | 0.04912 | -0.70876006 | Four and a half LIM domains 1 |
| 219941_at | TMEM19 | 3.69E-03 | 0.09723 | -0.71112301 | Transmembrane protein 19 |
| 201109_s_at | THBS1 | 1.09E-03 | 0.05351 | -0.7128624 | Thrombospondin 1 |
| 202534_x_at | DHFR | 7.34E-04 | 0.07779 | -0.71973803 | Dihydrofolate reductase |
| 220026_at | CLCA4 | 2.15E-04 | 0.03106 | -0.71989455 | Chloride channel accessory 4 |
| 61734_at | RCN3 | 1.22E-03 | 0.05602 | -0.72014743 | Reticulocalbin 3 |
| 202007_at | NID1 | 4.26E-04 | 0.03778 | -0.72303995 | Nidogen 1 |
| 207714_s_at | SERPINH1 | 1.08E-03 | 0.05351 | -0.72508596 | Serpin family H member 1 |
| 217771_at | GOLM1 | 1.81E-04 | 0.0302 | -0.72509919 | Golgi membrane protein 1 |
| 201147_s_at | TIMP3 | 2.59E-04 | 0.03216 | -0.72538653 | TIMP metallopeptidase inhibitor 3 |
| 219209_at | IFIH1 | 1.67E-03 | 0.06569 | -0.72661232 | Tnterferon induced with helicase C domain 1 |
| 202311_s_at | COL1A1 | 1.01E-05 | 0.01375 | -0.73470747 | Collagen type I alpha 1 chain |
| 215236_s_at | PICALM | 5.61E-04 | 0.04128 | -0.73751159 | Phosphatidylinositol binding clathrin assembly protein |
| 208654_s_at | CD164 | 1.85E-05 | 0.01884 | -0.74420569 | CD164 molecule |
| 218039_at | NUSAP1 | 1.28E-03 | 0.09711 | -0.75079306 | Nucleolar and spindle associated protein 1 |
| 48808_at | DHFR | 3.58E-04 | 0.06192 | -0.75662488 | Dihydrofolate reductase |
| 211672_s_at | ARPC4-TTLL3///ARPC4 | 3.69E-04 | 0.0369 | -0.76176808 | ARPC4-TTLL3 readthrough///actin related protein 2/3 complex subunit 4 |
| 219684_at | RTP4 | 6.47E-06 | 0.01603 | -0.7632737 | Receptor transporter protein 4 |
| 202465_at | PCOLCE | 1.52E-04 | 0.02898 | -0.76507134 | Procollagen C-endopeptidase enhancer |
| 200672_x_at | SPTBN1 | 6.49E-04 | 0.04306 | -0.76981934 | Spectrin beta, non-erythrocytic 1 |
| 220988_s_at | C1QTNF3 | 2.27E-03 | 0.07709 | -0.77386042 | C1q and tumor necrosis factor related protein 3 |
| 212009_s_at | STIP1 | 1.24E-04 | 0.02759 | -0.77877232 | Stress induced phosphoprotein 1 |
| 216971_s_at | PLEC | 2.35E-03 | 0.07834 | -0.7812013 | Plectin |
| 216598_s_at | CCL2 | 4.54E-04 | 0.06737 | -0.78616452 | C-C motif chemokine ligand 2 |
| 221730_at | COL5A2 | 9.86E-06 | 0.01375 | -0.79044276 | Collagen type V alpha 2 chain |
| 202446_s_at | PLSCR1 | 5.99E-04 | 0.04186 | -0.7960742 | Phospholipid scramblase 1 |
| 207542_s_at | AQP1 | 4.33E-05 | 0.01924 | -0.79804846 | Aquaporin 1 (Colton blood group) |
| 211559_s_at | CCNG2 | 7.63E-05 | 0.02104 | -0.80320592 | Cyclin G2 |
| 204426_at | TMED2 | 5.71E-05 | 0.01924 | -0.80872176 | Transmembrane p24 trafficking protein 2 |
| 212667_at | SPARC | 4.06E-04 | 0.03778 | -0.80900018 | Secreted protein acidic and cysteine rich |
| 204284_at | PPP1R3C | 2.97E-05 | 0.01884 | -0.81815573 | Protein phosphatase 1 regulatory subunit 3C |
| 203675_at | NUCB2 | 1.58E-05 | 0.0176 | -0.82240159 | Nucleobindin 2 |
| 210756_s_at | NOTCH2 | 1.00E-04 | 0.02477 | -0.82613569 | Notch 2 |
| 202086_at | MX1 | 2.94E-03 | 0.08811 | -0.82889492 | MX dynamin like GTPase 1 |
| 205226_at | PDGFRL | 1.63E-03 | 0.06471 | -0.83491498 | Platelet derived growth factor receptor like |
| 215707_s_at | PRNP | 2.63E-03 | 0.08352 | -0.83727819 | Prion protein |
| 214453_s_at | IFI44 | 2.74E-03 | 0.08569 | -0.84186913 | Interferon induced protein 44 |
| 208047_s_at | NAB1 | 4.89E-06 | 0.0099 | -0.85842177 | NGFI-A binding protein 1 |
| 211959_at | IGFBP5 | 3.77E-04 | 0.06365 | -0.86720103 | Insulin like growth factor binding protein 5 |
| 217430_x_at | COL1A1 | 5.75E-05 | 0.01924 | -0.87503584 | Collagen type I alpha 1 chain |
| 209896_s_at | PTPN11 | 6.80E-04 | 0.0441 | -0.88274411 | Protein tyrosine phosphatase, non-receptor type 11 |
| 201842_s_at | EFEMP1 | 8.36E-05 | 0.04447 | -0.9054224 | EGF containing fibulin like extracellular matrix protein 1 |
| 212143_s_at | IGFBP3 | 2.86E-03 | 0.08736 | -0.91225297 | Insulin like growth factor binding protein 3 |
| 204439_at | IFI44L | 4.60E-04 | 0.06737 | -0.92971193 | Interferon induced protein 44 like |
| 203666_at | CXCL12 | 1.11E-03 | 0.05377 | -0.94859903 | C-X-C motif chemokine ligand 12 |
| 213068_at | DPT | 9.01E-05 | 0.02324 | -0.99002783 | Dermatopontin |
| 201617_x_at | CALD1 | 1.63E-03 | 0.06471 | -0.99397639 | Caldesmon 1 |
| 214059_at | IFI44 | 5.68E-05 | 0.01924 | -1.0072761 | Interferon induced protein 44 |
| 205200_at | EXOSC7///CLEC3B | 1.51E-03 | 0.06204 | -1.0119182 | Exosome component 7///C-type lectin domain family 3 member B |
| 201291_s_at | TOP2A | 1.48E-04 | 0.05454 | -1.04167567 | Topoisomerase (DNA) II alpha |
| 207977_s_at | DPT | 2.24E-05 | 0.01884 | -1.08316103 | Dermatopontin |
| AFFX-HSAC07/X00351_5_at | ACTB | 1.18E-04 | 0.02729 | -1.10100279 | Actin beta |
| 214059_at | IFI44 | 2.54E-05 | 0.03165 | -1.13929758 | Interferon induced protein 44 |
| AFFX-HUMISGF3A/M97935_MB_at | STAT1 | 4.38E-05 | 0.01924 | -1.17645276 | Signal transducer and activator of transcription 1 |
| 202765_s_at | FBN1 | 5.31E-05 | 0.01924 | -1.20508855 | Fibrillin 1 |
| 217294_s_at | ENO1 | 4.93E-04 | 0.03887 | -1.23764645 | Enolase 1 |
| 204439_at | IFI44L | 6.54E-04 | 0.0431 | -1.31327283 | Interferon induced protein 44 like |
| 204427_s_at | TMED2 | 7.47E-05 | 0.02104 | -1.34133271 | Transmembrane p24 trafficking protein 2 |
| 213797_at | RSAD2 | 2.09E-03 | 0.07396 | -1.36642994 | Radical S-adenosyl methionine domain containing 2 |
| 209687_at | CXCL12 | 3.38E-04 | 0.03527 | -1.45006464 | C-X-C motif chemokine ligand 12 |

## Supplementary Figures


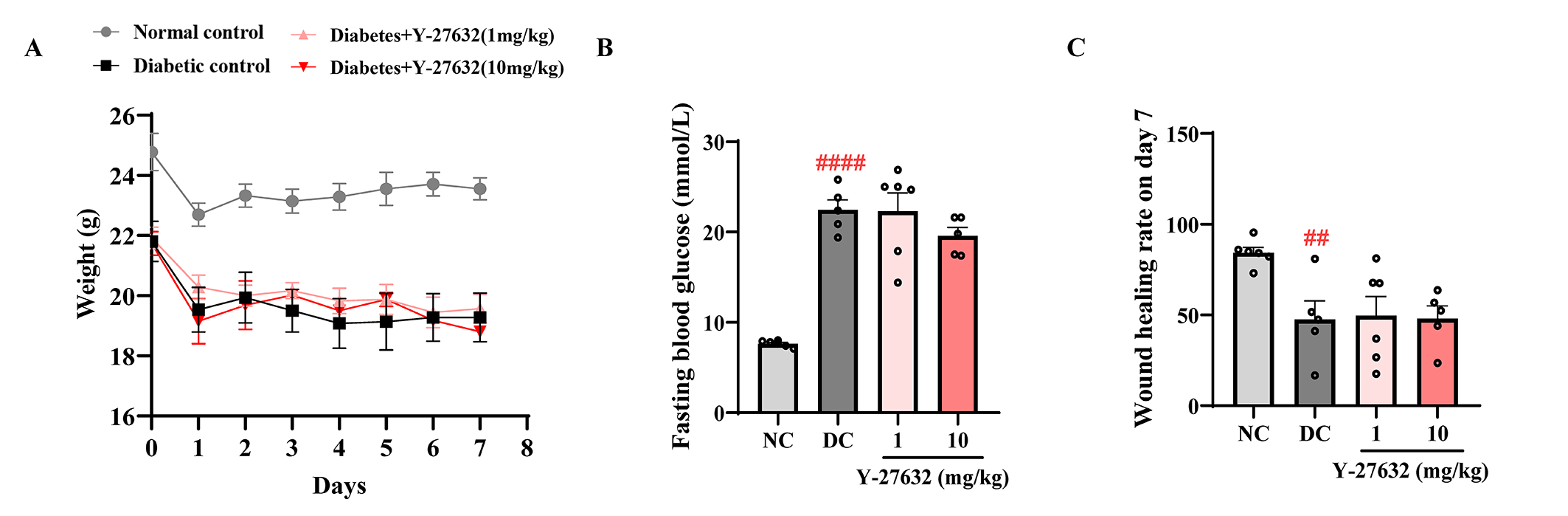


**Supplementary Figure 1.** The effect of CG-930 on T1D mice. (A) Body weight. (B) Fasting blood glucose on day 7. (C) Wound healing rate on day 7. Data presented are individual values with means ± SEM from n = 6 for each group. Statistical analysis tested by one-way ANOVA and two-way ANOVA (figure A). ## *p* < 0.01 and #### *p* < 0.0001 vs. the NC group. * *p* < 0.05 vs. the DC group.


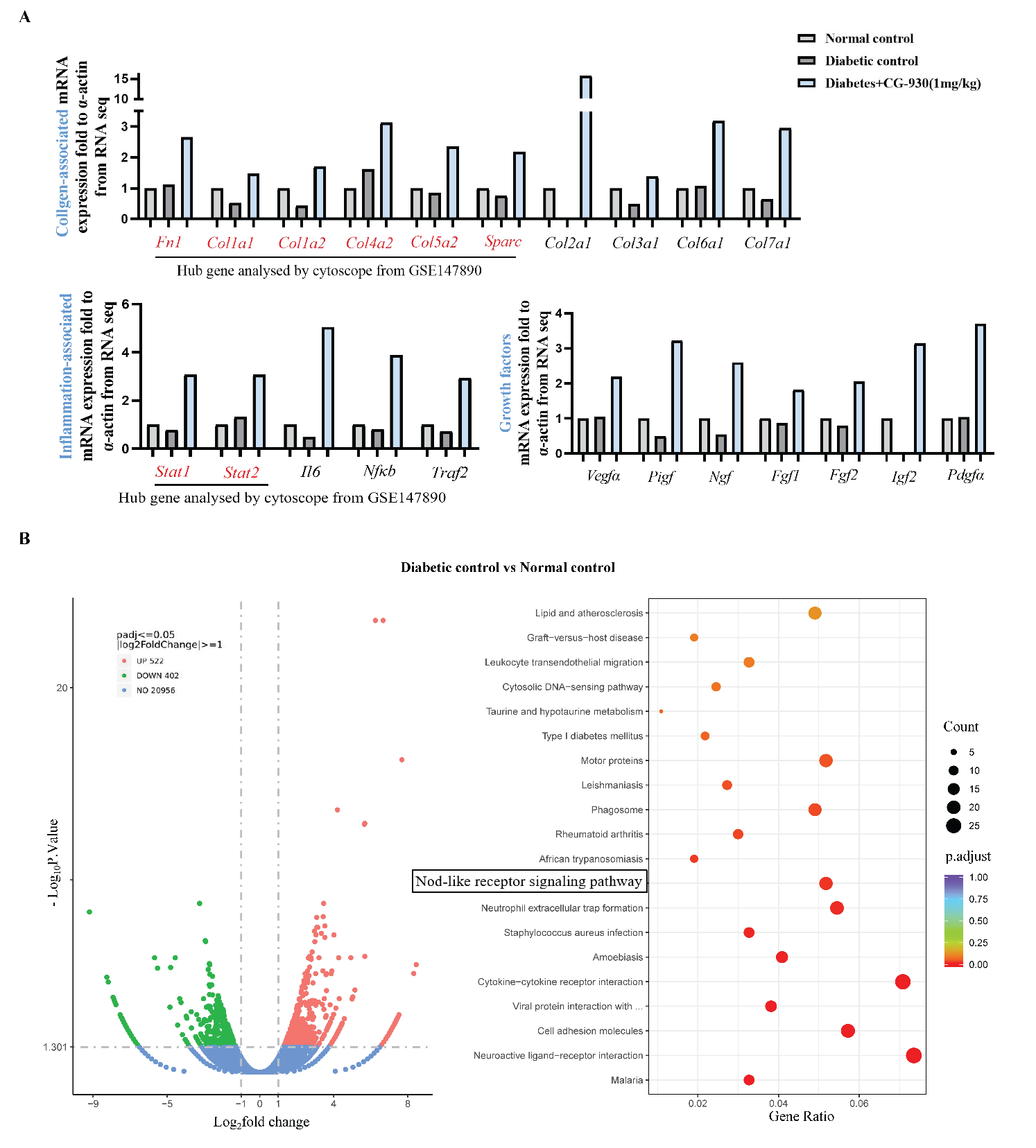


**Supplementary Figure 2.** Study on the mechanism of CG-930. (A) Relative mRNA expression of genes related to collagen synthesis, inflammation, and growth factors from RNA sequencing. (B) Volcano plot and KEGG dotplot of DEGs between DC and NC wounds.
