# Supplementary figures and images for: Identification of compounds to promote diabetic wound healing based on transcriptome signature
Source: Front Pharmacol. 2025 Jun 2;16:1576056. doi: 10.3389/fphar.2025.1576056 (PMC12171139; doi:10.3389/fphar.2025.1576056)

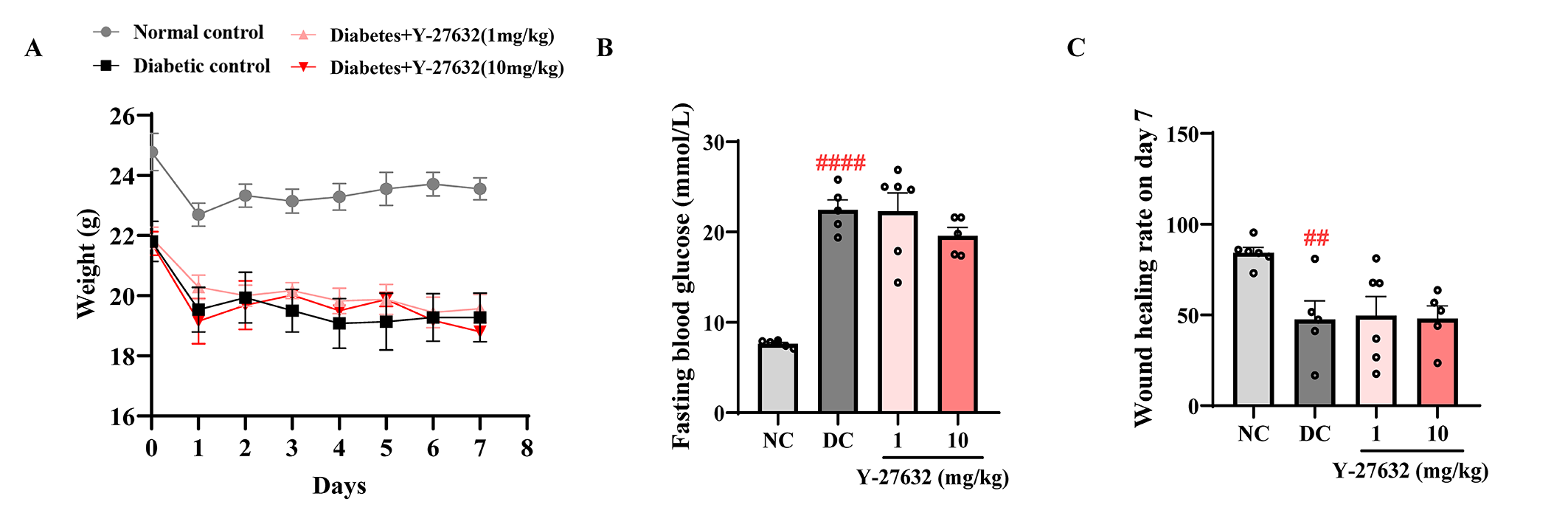

Supplement: Supplementary file 1 [file DataSheet1.zip › Supplementary material/2. Supplementary figure/Supplementary figure 1.tif]
